# DRPPM-PATH-SURVEIOR: Plug-and-Play Survival Analysis of Pathway-level Signatures and Immune Components

**DOI:** 10.21203/rs.3.rs-2688545/v1

**Published:** 2023-03-14

**Authors:** Alyssa Obermayer, Darwin Chang, Gabrielle Nobles, Mingxiang Teng, Aik-Choon Tan, Xuefeng Wang, Steven Eschrich, Paulo Rodriguez, G Daniel Grass, Soheil Meshinchi, Ahmad Tarhini, Dung-tsa Chen, Timothy Shaw

**Affiliations:** H. Lee Moffitt Cancer Center and Research Institute; H. Lee Moffitt Cancer Center and Research Institute; H. Lee Moffitt Cancer Center and Research Institute; H. Lee Moffitt Cancer Center and Research Institute; Huntsman Cancer Institute, University of Utah, Salt Lake City, UT; H. Lee Moffitt Cancer Center and Research Institute; H. Lee Moffitt Cancer Center and Research Institute; H. Lee Moffitt Cancer Center and Research Institute; H. Lee Moffitt Cancer Center and Research Institute; Fred Hutchinson Cancer Research Center, Seattle, WA; H. Lee Moffitt Cancer Center and Research Institute; H. Lee Moffitt Cancer Center and Research Institute; H. Lee Moffitt Cancer Center and Research Institute

**Keywords:** Pathway-level survival analysis, R Shiny app, hazard ratio GSEA, pathway clustering

## Abstract

Pathway-level survival analysis offers the opportunity to examine molecular pathways and immune signatures that influence patient outcomes. However, available survival analysis algorithms are limited in pathway-level function and lack a streamlined analytical process. Here we present a comprehensive pathway-level survival analysis suite, DRPPM-PATH-SURVEIOR, which includes a Shiny user interface with extensive features for systematic exploration of pathways and covariates in a Cox proportional-hazard model. Moreover, our framework offers an integrative strategy for performing Hazard Ratio ranked Gene Set Enrichment Analysis (GSEA) and pathway clustering. As an example, we applied our tool in a combined cohort of melanoma patients treated with checkpoint inhibition (ICI) and identified several immune populations and biomarkers predictive of ICI efficacy. We also analyzed gene expression data of pediatric acute myeloid leukemia (AML) and performed an inverse association of drug targets with the patient’s clinical endpoint. Our analysis derived several drug targets in high-risk KMT2A-fusion-positive patients, which were then validated in AML cell lines in the Genomics of Drug Sensitivity database. Altogether, the tool offers a comprehensive suite for pathway-level survival analysis and a user interface for exploring drug targets, molecular features, and immune populations at different resolutions.

## 1. Background

Organizing biological knowledge into pathways facilitates the integrative analysis of gene expression data derived from RNA sequencing and proteomics profiling. Common pathway-level analysis tools, such as ENRICHR[1], GSEA [2], are able to perform pathway enrichment analysis based on gene set databases (e.g., KEGG [3], REACTOME [4], MSIGDB [5], LINCS1000 [6], and the Cell Marker database [7]). While these pathway analysis tools tend to focus on differentially expressed genes between two groups of samples, an alternative approach is to infer a pathway activity score in a single sample by transforming the expression of a set of genes into a single value using gene summary statistics, such as maxmean statistics [8], PLAGE [9], GSVA [10], and ssGSEA [2, 11]. Based on this approach, scores derived from custom gene sets or from network analyses [12–14] can then be used to dichotomize the patient population for survival analysis. For example, PERK-associated gene activity was found to be associated with a higher risk in melanoma patients [15], RAS dependency index was developed in pancreatic adenocarcinoma [16], LCK network activity was associated with T-cell acute lymphoblastic leukemia patient survival [17], and an epithelial-mesenchymal transition score was found to be associated with poorer disease-free survival in ovarian and colorectal patients [18]. Moreover, single scores derived from immune markers can be used as an estimate of immune components (e.g., Xcell [19], TIMER 2.0 [20], and geometric mean estimation of tumor infiltrative leukocytes [21]). These immune scores can then be applied in cancer patient classification [22], as a biomarker of check-point immunotherapy response [23], or as a prognosis marker that’s predictive of clinical outcome [24].

These integrative summary scores represent a useful approach in highlighting signaling pathways and immune populations that correlate with the clinical outcome. But existing survival analysis tools either lack a user-friendly interface or has limited functionality for systematic screening of large pathway databases. They are often restricted in available patient cohort or limited to a small subset of pathways [25–27] and are often incapable of accepting external user input data [25–28] or clinically relevant covariates [26, 29, 30]. Thus, to facilitate the public mining of retrospective clinical studies, we introduce DRPPM-PATH-SURVEIOR, a comprehensive plug-and-play suite for pathway-level survival analysis of signature databases. Our tool is presented with the following unique features:

1. A one-stop tool for expression-based survival analyses.
2. The ability to include multiple covariates inside the Cox-proportion hazard pathway model.
3. The ability to summarize prioritized gene signatures into relevant clusters and pathway modules.
4. The ability to perform hazard ratio ranked gene set enrichment analysis

Altogether, survival analysis is a critical branch of statistics for analyzing the time-to-event, and our tool facilitates a comprehensive survival analysis of pathway-level scores (an additional comparison of features is available in the **Supplementary Result Section**).

## 2. Implementation

### Overview of the entire pipeline.

**Figure 1:**
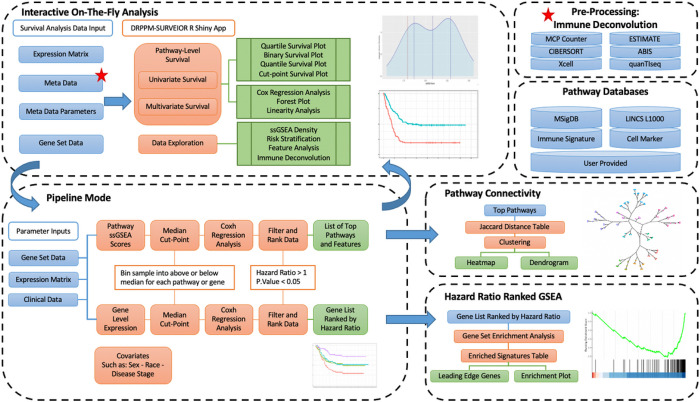
Schematic workflow of DRPPM-PATH-SURVEIOR: a pathway-level analysis suite. In the Shiny interface (Top Left), users can provide a gene expression matrix matched with patient outcome meta information and a custom gene-set pathway. The shiny App will calculate a score for the selected pathway using single sample gene set enrichment (ssGSEA) and dichotomized based on the median, optimum cut-point, quartiles, or user-specified cut-point. A univariate Cox hazard regression analysis can be performed. Additional patient features or immune deconvoluted features (Red Star Top Right) can be incorporated into a multivariate Cox hazard model and explored by our interface. In the Pipeline mode (Bottom Left), these scores were further dichotomized above and below the median ssGSEA score. A Cox regression analysis can be performed on each gene set to generate a comprehensive table of gene sets to filter according to significant, high-risk patients (hazard ratio > 1, p.value < 0.05). A hazard ratio ranked gene list can be analyzed by GSEA (Bottom Right), and prioritized pathways can be visualized by pathway connectivity quantified by the Jaccard Index (Middle Right).

DRPPM-PATH-SURVEIOR is implemented in the R environment, and packages will be automatically installed during runtime. There are four major components of the DRPPM-PATH-SURVEIOR (Fig. 1), which include: 1) The Interactive (UI) Mode. This feature allows for a point-and-click pathway survival analysis. The interactive mode offers the ability to perform select immune deconvolution in real time and perform univariate or complex multivariate analyses of clinical features. 2) The Pipeline (Advanced) Mode. This feature performs a complete survival analysis of pathway databases and gene features. Covariates can be included as part of the systematic screening, and the P-values are corrected by Benjamini-Hochberg. 3) Pathway Connectivity. This feature allows the user to evaluate the similarity between pathways and group pathways that are predictive of the clinical outcome. 4) Hazard Ratio Ranked Gene Set Enrichment Analysis (GSEA). This user interface performs a GSEA analysis based on the gene-level hazard ratio ranking derived from the Pipeline Mode. This feature facilitates the identification of clinically relevant pathways and, in turn, identifies regulators that can drive the underlying gene expression. Additional installation and usage instructions is available in the **Supplementary Method Section.**

### “On-the-fly” Mode: Shiny Interface

DRPPM-PATH-SURVEIOR is a comprehensive framework for analyzing and visualizing “on-the-fly” associations of immune signatures and pathways scores with a clinical endpoint. The application facilitates the partitioning of patients based on pathway scores, estimated immune cells, and gene expression level, followed by univariate Cox-regression survival analysis and multivariate Cox-regression analysis. DRPPM-PATH-SURVEIOR uses several R packages, including survival (v3.2–11), survminer (v0.4.9), GSVA (v1.40.1) [10], and immunedeconv [31](v2.1.0). Pathway score is calculated with the gsva() function based on ssGSEA, GSVA, plage, or zscore. Immune deconvolution is performed with the immunedeconv R package, which includes several deconvolution packages, such as CIBERSORT [32], ESTIMATE [33], and MCP counter [34]. For multivariate analysis, a covariate can be selected from the user-provided meta-information file. The multivariate survival analysis can be performed through additive and multiplicative interaction of two or more variables.

To evaluate the association between pathway and survival S over time t,S(t), is defined through hazard function, h(t), as

$$S(t)=exp(-\int_{0}^{t}\;h(x)dx)$$

$$h(t)=h_{0}(t)\times e^{\left(\beta_{1}\times X_{1}\right)}$$

where h(t) is a hazard rate at time t and $h_{0}(t)$ is the baseline hazard rate, $\beta_{1}$ is the coefficient related to hazard with $\beta_{1}>0$ as high risk and $\beta_{1}<0$ as low risk for $X_{1}$, a dichotomized based on the gene or pathway score.

To evaluate the pathway association with survival after adjusting for patient meta information $X_{2}$ is defined as

$$h(t)=h_{0}(t)\times e^{\left(\beta_{1}\times X_{1}+\beta_{2}\times X_{2}\right)}$$

To evaluate the associated interaction between the pathway and patient meta information is defined by $\beta_{3}$ as

$$h(t)=h_{0}(t)\times e^{\left(\beta_{1}\times X_{1}+\beta_{2}\times X_{2}+\beta_{3}\times X1\times X_{2}\right)}$$

### Pipeline Mode: Systematic Pathway-level Survival Analysis

To facilitate the identification of top high-risk pathways and genes, we have developed a pipeline to systematically assess pathways associated with hazard by a Cox proportional hazard function. The user can provide or select individual genes and pathway databases to perform a systematic screening. Each expression feature is stratified based on a median cutoff. The user also has the option of performing a systematic screening with the inclusion of a covariate as an additive or multiplicative interactive model. The p.value is calculated on the likelihood ratio, wald test. An adjusted p.values can be calculated based on Benjamini-Hochberg correction method. In the output table, genes and pathways are ranked by the likelihood ratio p.value.

### Connectivity Mode: Pathway Gene-set Connectivity

The Connectivity Mode offers the user the ability to analyze the similarity between pathways associated with survival. The hazard ratio ranked pathways from the pipeline mode can be used as input to the DRPPM-Jaccard-Connectivity R Shiny app to generate hierarchical clustering based on gene-set similarity. A Jaccard function can calculate distance between pathways:

The Jaccard score function J for two pathways A and B is defined as

J(A,B)=|A∩B|/|A∪B|

where

A contains n set of genes, A = [a1, a2, …, an]

B contains m set of genes, B = [b1, b2, …, bm]

The Jaccard matrix can be visualized as a heatmap.

Next, the pathways can be clustered using the hclust function from R (v4.1.0) into k-groups (user-specified). Clusters can be visualized as a dendrogram. To overlap survival-associated gene expression, genes within the pathway can be displayed as a table with a flexible sorting feature and added annotation information.

### GSEA Mode: Hazard Ratio Ranked Gene Set Enrichment Analysis

From the pipeline mode, we can derive a hazard ratio ranked gene list, which can then be applied as input to the DRPPM-HazardRatio-Ranked-GSEA R Shiny app. This application takes a two-column file of the gene symbols and hazards ratios, which is used as input to the GSEA function from clusterProfiler (v4.0.5). The application performs GSEA, and results can be visualized as a table with additional options for visualizing the GSEA plots through the gseaplot2 function from enrichplot (v1.12.3).

## 3. Results

To demonstrate functionalities of the DRPPM-PATH-SURVEIOR framework, we have included use-case examples of biomarker discovery in a cohort of immunotherapy-treated melanoma patients. We have also provided an example use-case strategy for drug repurposing in pediatric acute myeloid leukemia patients.

### Identifying Immune Pathways Associated with Effective Checkpoint Inhibition Treatment

**Figure 2:**
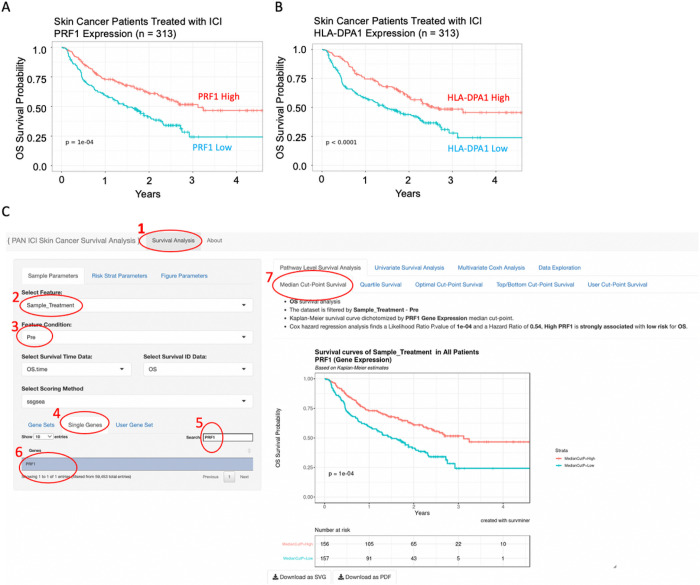
Overall survival curves of immunotherapy treated skin cancer patients. Overall survival curves of immunotherapy treated skin cancer patients dichotomized based on perforin 1 (PRF1) (A) and HLA-DPA1 (B). User interface of the gene-level survival analysis of PRF1. Samples were filtered based on pre-treatment (2 and 3). PF1 was searched and selected from the Single-gene list (4, 5, 6). Kaplan meier curve is shown comparing a median dichotemized patient population (7).

**Figure 3:**
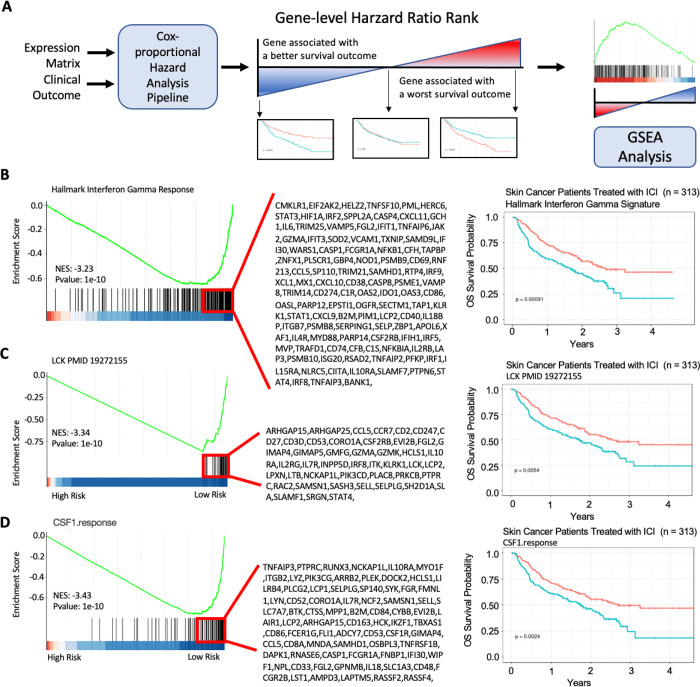
Gene-set Enrichment Analysis based on Genes Ranked by Hazard Ratio. **A)** Genes can be ranked based on the hazard ratio for overall survival (OS). GSEA can be applied to examine for similarity in gene features that are shared in high or low-risk diseases. Interferon Gamma (**B)**, the LCK Signature **(C)**, and the CSF1 response signature **(D)** were found to be associated with low-risk patients based on GSEA. Kaplan Meier curves showing patients dichotomized based on ssGSEA immune signatures are shown on the right.

**Figure 4:**
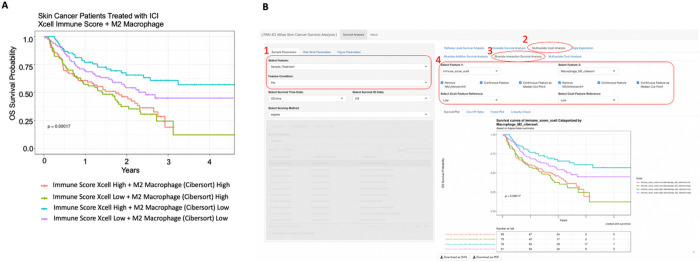
Associating Immune Scores with Patient Survival. **A)** Skin Cancer Patients treated with ICI dichotemized based on xCell derived Immune Scores as well as M2 Macrophage estimated from Cibersort. Patients with High Immune Score and Low M2 Macrophage were associated with better survival. **B)** User Interface that access this function from the Shiny App is 1) Select Feature Condition to restrict to pretreated patients, 2) Multivariate Coxh Analysis, 3) Bivariate Interaction Survival Analysis, 4) Select Immune Score (on left) and M2 Macrophage (on right). The feature can be dichotemized “on-the-fly” if it is a continuous variable (4).

**Figure 5:**
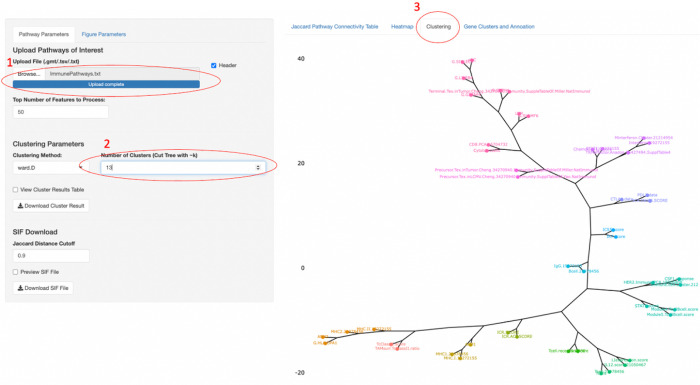
Pathway connectivity Analysis User Interface. 1) Input list of pathway that satisfy the selection criterion. 2) Set the number of clusters captured by the algorithm. 3) Select the Clustering Visualization Tab.

To identify predictive biomarkers that facilitate patient selection of patients suitable for immune checkpoint inhibitor (ICI) treatment, we integrated 313 melanoma patients treated with ICI from Riaz et al. (n = 51) [35], Hugo et al. (n = 25) [36], Van Allen et al. (n = 25) [37](n = 42), Liu et al. (n = 122) [38], and Gide et al. (n = 73) [39]. First, we performed a systematic univariate Cox-hazard analysis of individual gene expression in the “**Pipeline Mode**” and identified 100 genes associated with the better prognosis (**Supplementary Table S1**). These include PRF1 and HLA-DPA1, which have been previously reported as predictive biomarkers for ICI therapy [40] (Fig. 2). “**On-the-fly analysis mode**” further demonstrate PRF1 and HLA-DPA1 were significantly higher in patients who respond to ICI treatment (**Supplementary Figure S1**). We then ranked the genes based on hazard ratio derived from the cox-proportion hazard model and performed a **Hazard Ratio Ranked GSEA analysis** of the Hallmark database (Fig. 3A). Interferon Gamma was found associated with Low-risk patients (Fig. 3B). Consistently, immune signatures associated with LCK and CSF1 were also associated with Low-risk patients (Fig. 3C, 3D). Through immune deconvolution, we derived an immune score from xCell [19] and an estimated M2-like macrophage population from Cibersort [32]. We found that high immune infiltration with low M2-like (immune suppressive) macrophages were associated with better outcome (Fig. 4A, 4B). Next, we used the “Pipeline Mode” to perform a systematic univariate Cox-hazard analysis of immune signatures and identified to identify 69 immune signatures associated with a better outcome (**Supplementary Table S2**). A systematic assessment of immune pathway followed by **Pathway Connectivity analysis** demonstrated 13 immune modules captured a favorable outcome in pretreated RNA samples, including CD8 cytotoxicity, antigen presentation, interferon and immune checkpoint marker signatures (Fig. 5, **Supplementary Figure S2**). Altogether, our tool provides suggests favorable outcome and is linked with immune activation.

### Survival-Directed Therapeutic Discovery in Acute Myeloid Leukemia

**Figure 6:**
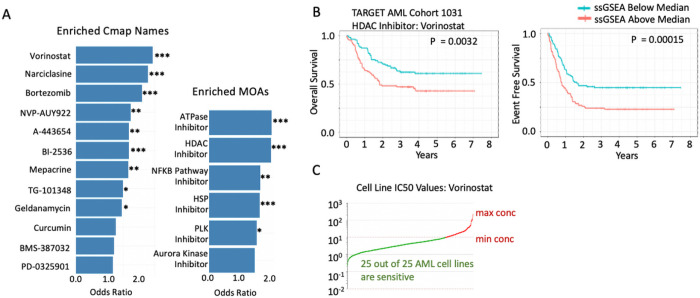
Overall and event free survival curves of KMT2A positive patients in TARGET AML 1031. **A)** After performing the LINC1000 screening of drug targets in AML, the “High Risk” gene sets were further prioritized by counting the number of drug target gene sets, which was further categorized based on mechanism of action (MOA) to identify enriched drug targets in high-risk patients. Drug and MOA were evaluated for enrichment using a Fisher’s exact test. **B)** Overall and event-free survival curves of KMT2A positive patients in TARGET AML 1031. Patients are classified by the ssGSEA score derived from genes affected by the Vorinostat. **C)** Cell-line sensitivity ranking based on IC50 values of Vorinostat from GDSC

To leverage our framework for therapeutic discovery, we obtained the gene expression data and clinical annotation of 220 patients with the KMT2A fusion event from the National Cancer Institute TARGET pediatric acute myeloid leukemia (AML) 1031 cohort (0–22 years of age). The translocation event of the gene KMT2A, also known as mixed lineage leukemia (MLL), is frequently identified in pediatric AML. Through its multiple fusion partners arises a diverse patient population with a need for advanced risk stratification [43]. Through the DRPPM-PATH-SURVEIOR suite of tools, we examined pathways and genes associated with poor outcome and identify therapeutic targets in high-risk patients. First, single samples gene set enrichment analysis (ssGSEA) was performed using the expression data in tandem with the Library of Integrated Network-based Cellular Signatures (LINCS; 31,028 gene sets) LINCS1000 gene sets to calculate the pathway scores (**Supplementary Figure S3**). Next, the patients were dichotomized through a median cut-point of each pathway score into an above-median or below median group, followed by a Cox proportional hazards regression using the patient’s overall survival (OS) and event free survival (EFS). A hazard ratio value greater than one and a P-value less than 0.05 was applied to identify significant pathways associated with high risk. To prioritize a putative therapeutic target that downregulates genes associated with high-risk AML in the KMT2A subgroup, we examined enriched connectivity map (cMAP) name and Mechanism of Action. We found 12 enriched Cmap names and 6 enriched drug categories grouped by their mechanism of action (Fig. 6A). Notably, genes downregulated by the HDAC inhibitor, Vorinostat, were associated with the worst prognosis based on OS and EFS (Fig. 6B). Furthermore, Vorinostat was highly sensitive in KMT2A fusion-positive AML cell lines based on the genomics of drug sensitivity in cancer (GDSC) database (Fig. 6C). Taken together, we presented an integrative strategy utilizing DRPPM-PATH-SURVEIOR to prioritize pathways based on patient risk and identified a known therapeutic target in high-risk KMT2A fusion-positive AML patients.

## 4. Conclusion

DRPPM-PATH-SURVEIOR is designed to visualize and perform systematic survival analysis based on gene pathway information. The application is designed for users with limited experience in programming as well as for advanced users to perform systematic high-throughput pathway screening. In the interactive mode, the Shiny application will ensure reproducibility and can be easily set up and applied in any cohort. In the pipeline mode, the user can apply univariate and multivariate analysis of pathway and patient covariates associated with patient outlook. Our current application can also perform GSEA based on hazard ratio ranking as well as pathway clustering to examine shared gene and pathway features associated with survival. As more RNA sequencing and proteomics data are being captured in large clinical trials, we anticipate DRPPM-PATH-SURVEIOR will enable a collaborative environment for exploring pathway-level and immune features that is predictive of treatment efficacy, especially for immunotherapy.

## List Of Abbreviations

AML: Acute Myeloid Leukemia
GSEA: Gene Set Enrichment Analysis
GDSC: Drug sensitivity in cancer database
ICI: Immune checkpoint inhibitor
OS: Overall survival
EFS: Event free survival
CMAP: Connectivity Map
MOA: Mechanism of Action
TARGET: Therapeutically Applicable Research To Generate Effective Treatments
